# Prediction of Storage Quality and Multi-Objective Optimization of Storage Conditions for Fresh *Lycium barbarum* L. Based on Optimized Latin Hypercube Sampling

**DOI:** 10.3390/foods14162807

**Published:** 2025-08-13

**Authors:** Xiaobin Mou, Xiaopeng Huang, Guojun Ma, Qi Luo, Xiaoping Yang, Shanglong Xin, Fangxin Wan

**Affiliations:** College of Mechanical and Electronical Engineering, Gansu Agricultural University, Lanzhou 730070, China; mouxb@gsau.edu.cn (X.M.); huangxp@gsau.edu.cn (X.H.);

**Keywords:** fresh wolfberry, quality characteristic, the initial maturity, storage temperature, storage time, neural network model, multi-objective optimization

## Abstract

Quality control of fresh *Lycium barbarum* during storage presents significant challenges, particularly regarding the unclear relationship between quality characteristics and storage conditions. This study analyzes the changes in qualitative and structural characteristics, including fruit hardness, soluble solid content (SSC), titratable acidity (TA), and vitamin C (Vc), under various storage conditions (temperature, duration, and initial maturity). We employed optimized Latin hypercubic sampling to develop radial basis function neural networks (RBFNNs) and Elman neural networks to establish predictive models for the quality characteristics of fresh wolfberry. Additionally, we applied the Particle Swarm Optimization (PSO) algorithm to determine the optimal solution for the constructed models. The results indicate a significant variation in how different storage conditions affect the quality characteristics. The established RBFNN predictive model exhibited the highest accuracy for TA and Vc during the storage of fresh wolfberry (R^2^ = 0.99, RMSE = 0.21 for TA; R^2^ = 0.99, RMSE = 0.19 for Vc), while the predictive performance for hardness and SSC was slightly lower (R^2^ = 0.98, RMSE = 385.78 for hardness; R^2^ = 0.94, RMSE = 2.611 for SSC). Multi-objective optimization led to the conclusion that the optimal storage conditions involve harvesting *Lycium barbarum* fruits at an initial maturity of 60% or greater and storing them for approximately 10 days at a temperature of 10 °C. Under these conditions, the fruit hardness was observed to be 15 N, with SSC at 17.5%, TA at 1.22%, and Vc at 18.5 mg/100 g. The validity of the prediction model was confirmed through multi-batch experimental verification. This study provides theoretical insights for predicting nutritional quality and informing storage condition decisions for other fresh fruits, including wolfberries.

## 1. Introduction

*Lycium barbarum* L., an important economic crop known for its dual purpose as a source of medicine and food (strong market demand in the European Union and North America [1]), faces significant post-harvest losses due to the thin skin, high sugar content, and high water content of its fresh fruits. These fruits experience concentrated ripening during the summer and fall, leading to vigorous respiratory metabolism that makes them susceptible to corruption and deterioration [2]. Furthermore, the existing industry lacks a theoretical framework for the quality control of fresh fruit storage, highlighting the urgent need to establish a scientifically based system for freshness preservation technologies.

The current research is subject to several limitations. First, there is a restriction in the scope of the study, as existing findings predominantly focus on the drying technology of wolfberry. Consequently, there is a significant lack of research on the quality dynamics of fresh fruits during the storage period, including the mechanisms underlying the degradation of polysaccharides, vitamin C, and other active compounds. Second, methodological limitations arise from the use of traditional multiple linear regression (MLR), which is inadequate for addressing the nonlinear relationships between storage conditions and fruit quality [3]. Moreover, the application of artificial neural networks (ANNs) in the realm of fresh fruit preservation has thus far been limited to specific crops, such as kiwifruit [4] and jujube [5], with no comprehensive studies addressing goji berry polysaccharides (GBP). Despite the advantages of ANNs demonstrated in agricultural predictions—such as seed yield, fruit hardness, and electrical characteristics related to quality—the integration of ANNs with optimization algorithms, particularly in the context of enhancing goji berry storage, remains an unaddressed gap in the literature [6].

This paper employs an optimized Latin hypercube experimental design to systematically collect multidimensional storage data for fresh *Lycium barbarum* L. We construct a quality prediction model based on artificial neural networks (ANNs) to address the limitations of multiple linear regression (MLR) [3] while leveraging the advantages of ANNs in nonlinear approximation (e.g., winter jujube [5] and apple [7]) and biological system simulation [7,8]. Additionally, we perform multi-objective optimization through the introduction of the Particle Swarm Optimization (PSO) algorithm, drawing on the high accuracy (fitting rate > 95%) of PSO-BP in jujube [5]. For the first time, we identify the optimal storage parameter combinations for fresh wolfberry, contributing a universal freshness preservation program to the industry. Despite the advantages of ANNs in agricultural predictions (e.g., seed yield [9] and fruit hardness [10,11]), their integration with optimization algorithms for goji berry storage remains unexplored.

Therefore, this study aims to accomplish the following:

(i) To study the effect patterns of storage time, storage temperature, and fruit initial maturity on the hardness, SSC, TA, and Vc of fresh *Lycium barbarum* L. Miller during storage.
(ii) Using Latin hypercube sampling to develop neural networks, models based on radial basis function neural networks (RBFNNs) and Elman neural networks (ELMANs) were established to predict the quality characteristics of fresh *Lycium barbarum* L. based on storage environments. During the training of the neural networks, Latin hypercube sampling was employed to automatically identify the optimal hyperparameters of the neural networks, allowing for the analysis and comparison of the predictive accuracy of each model.
(iii) Based on the constructed predictive model, the contribution values of storage temperature parameters, storage time parameters, and fruit initial maturity parameters to hardness, soluble solid content (SSC), titratable acidity (TA), and vitamin C (Vc) content were analyzed.
(iv) Using the Particle Swarm Optimization (PSO) algorithm, the constructed predictive model was optimized for the values of hardness, soluble solid content (SSC), titratable acidity (TA), and vitamin C (Vc), as well as the storage condition parameters. This resulted in the determination of the optimal storage temperature and initial maturity parameter values suitable for the specified storage duration.

## 2. Materials and Methods

### 2.1. Materials

#### 2.1.1. Sample Selection and Determination of Initial Indicators

The fresh *Lycium barbarum* L. used in this experiment were sourced from the green and red varieties cultivated in the *Lycium barbarum* industrial park located in Shuanglong Township, Jingyuan County, Gansu Province, during July and August 2024. The fruits were handpicked from local farmers’ orchards, selecting only those exhibiting bright colors and a glossy appearance at each growth stage, while ensuring they were free of surface bruises. This selection process was critical for guaranteeing the accuracy and reliability of the experimental data. Experimental operations commenced within three hours post-harvest, with the fruits stored in a temperature-controlled environment at 4 °C. Furthermore, to ensure that each batch of selected *Lycium barbarum* L. met standard sample requirements and remained undamaged, only fruits with intact morphology, no signs of bruising, and normal physiological indices were chosen for testing. This meticulous selection aimed to facilitate a scientific and rigorous evaluation. The initial characteristics of the fresh *Lycium barbarum* L. measured in this experiment were as follows: single fruit weight 0.8 ± 0.1 g, length 1.5 ± 0.2 cm, hardness 18.1 ± 1.2 N, soluble solids content (SSC) 15.3 ± 1.2%, titratable acidity (TA) 2.1 ± 0.3%, and vitamin C (Vc) content 28.5 ± 2.1 mg/100 g (mean ± standard deviation, n = 100).

#### 2.1.2. Determination of Goji Berry Initial Maturity

The initial maturity of the samples was determined using the quantitative evaluation equation proposed by Lan et al. [12] for Korla pears. However, *Lycium barbarum* L., with the same growth patterns, may exhibit different quality due to variations in climatic conditions and field management at different production sites. Therefore, it is essential to calibrate the evaluation equation before assessing the initial maturity of the fruit. This method analyzes the initial maturity patterns of *Lycium barbarum* L. and utilizes the following equation to determine the trend of quality indicators during the maturation process of *Lycium barbarum* L.:

(1) $$M_{i}=\left|\frac{y_{i}-y_{1}}{y_{2}-y_{1}}\right|\times 100\%$$

where *M_i_* is the initial maturity, %; *y*_1_ is the initial value of the indices; *y*_2_ is the final value of the indices; and *y_i_* is the value of a particular index.

In this experiment, the variation ranges of hardness, SSC, TA, and Vc content were 659.85–2135.96 g, 5.37–23.54%, 0.295–3.49% and 9.75–35.16 mg/100 g, respectively. The initial maturity evaluation equation was calibrated according to Equations (2) and (3). No matter which is used (Equation (2) or Equation (3)), the results are identical for determining initial maturity.

(2) $$M_{SSC}=3.958-0.258y_{SSC}$$

(3) $$M_{Vc}=0.237y_{Vc}-0.434$$

where *M*_ssc_ is the harvest initial maturity determined by SSC, %o; *y*_ssc_ is the SSC value during harvest period, %; *M_Vc_* is the harvest initial maturity determined by Vc content, %; and *y_Vc_* is the *Vc* content value during harvest period, mg/100 g.

Equations (2) and (3) are mathematically equivalent formulations for calculating maturity, with Equation (2) based on SSC and Equation (3) on Vc. Both yield consistent maturity indices, as validated by experimental data (R^2^ = 0.98).

### 2.2. Methods

The storage experiment was conducted over a 30-day period in a controlled environment chamber maintained at constant temperature and humidity. The experiment encompassed eight temperature conditions: −4 °C, 0 °C, 4 °C, 8 °C, 12 °C, 16 °C, 20 °C, and 24 °C. For each combination of temperature and sampling time (every 12 h), three independent biological replicates (batches of fruit) were prepared and analyzed. Each replicate consisted of 50 fruits (total weight 40 ± 5 g), and quality measurements (hardness, SSC, TA, and Vc) were performed on 15 randomly selected fruits per replicate. Quality measurements for each replicate were conducted as described in the following sections.

The eight temperature conditions (−4 °C, 0 °C, 4 °C, 8 °C, 12 °C, 16 °C, 20 °C, and 24 °C) were selected based on (1) industry-relevant cold chain ranges (0–12 °C); (2) extreme scenarios (−4 °C for freezing effects; and 20–24 °C for ambient storage decay); and (3) the optimal temperature (8 °C) identified for nutrient retention in preliminary trials (Section 3.1).

Samples were collected every 12 h to monitor rapid physiological changes in this climacteric fruit, particularly respiration-driven quality shifts (e.g., SSC peak at Day 4; TA decline post-Day 8). High-frequency sampling ensures accurate decay curve modeling. All experiments were performed during July–August 2024, with exact sampling dates logged as raw data (available upon request).

#### 2.2.1. Determination of Hardness

**Figure 1:**
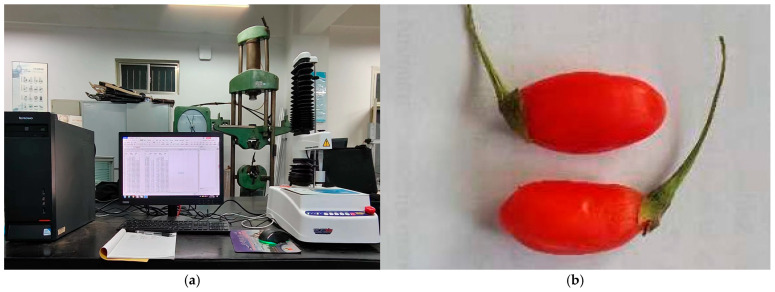
Texture parameter determination device: (**a**) *Lycium barbarum* L. compression experimental device; (**b**) part of the *Lycium barbarum* L. samples.

Texture parameters of *Lycium barbarum* L. chinense Miller were determined using the TPA (Texture Profile Analysis) method [13,14,15]. As shown in Figure 1, the test apparatus consisted mainly of a texture analyzer (c) and a computer. The test was carried out in 5 batches and tested within 5 d. The determination of the texture parameters of Chinese wolfberry from the same test batch was completed on the same day. During the test, the cut standard specimens were fixed in order under the flat plate of the physical property analyzer for the TPA test, and the P/35 compression probe was selected. Each test was carried out 3 times, and the average value was taken. The texture parameters measured in this experiment included hardness (N), which was obtained from the texture characteristic curves (Figure 1).

#### 2.2.2. Determination of SSC

The SSC serves as a key indicator for assessing the growth conditions and quality of crops. In wolfberry cultivation, monitoring SSC is essential for understanding the initial maturity and nutrient accumulation of the fruit, thereby providing a basis for determining optimal harvesting times and implementing effective fertilization management strategies. In this experiment, fruit pulp (2.000 ± 0.002 g) from equatorial regions was homogenized. SSC was measured via a PAL-1 refractometer (Atago, Tokyo, Japan) and expressed as °Brix” [16].

#### 2.2.3. Determination of TA

**Figure 2:**
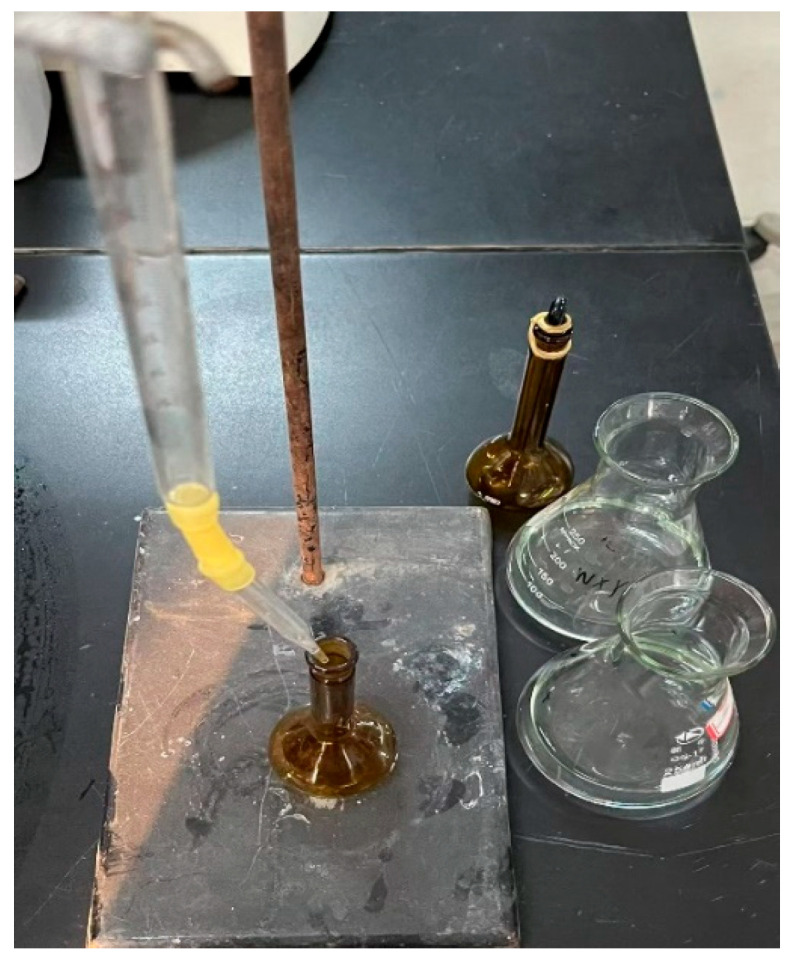
Titratable acid test device.

TA was determined by titrating 10 mL of fruit extract with 0.1 N NaOH to a pH 8.1 endpoint [17], expressed as % malic acid (Figure 2).

(4) $$TA=\left(\frac{V\times c\times (V_{1}-V_{0})\times f}{V_{s}\times m}\right)\times 100\%$$

In the formula, *V* represents the total volume of the sample extract in mL; *c* is the concentration of the NaOH titrant in mol/L; *V*_1_ is the volume of NaOH solution consumed in the titration of the filtrate in mL; *V*_0_ is the volume of NaOH solution consumed in the titration of distilled water in mL; *V_S_* is the volume of extract used for titration in mL; *m* is the mass of the sample in grams; and *f* is the conversion factor for malic acid in g/mol.

#### 2.2.4. Determination of Vc

Vc can represent the nutritional value of fruits, possessing antioxidant properties that delay the aging process of *Lycium barbarum* L. after harvesting and are associated with the prevention of chronic diseases in humans [18,19]. Following the method outlined in GB 5009.86—2016 [20], 20 g of *Lycium barbarum* L. at different maturities were weighed and ground into a homogeneous paste, then diluted to 200 mL with a 2% oxalic acid solution. A 10 mL aliquot was titrated with a standardized solution of 2,6-dichlorophenolindophenol until the solution remained pink for 15 s while simultaneously conducting a blank test.

#### 2.2.5. Statistical Analysis

Excel 2023 data analysis software (Microsoft, Redmond, WA, USA) was used for data processing and SPSS 25 software (IBM, Armonk, NY, USA) for one-way (ANOVA, New Providence, NJ, USA) analysis of variance, Duncan multiple comparisons and Pearson correlation analysis; Origin 2019 (Origin Lab, Northampton, MA, USA) software was used for image plotting and data regression analysis. Latin hypercube experiments and Predictive Model Fitting were used in combination with Isight Software(2023). A quality prediction model based on dielectric properties was constructed using MATLAB R2023a software, and training of the model was completed.

### 2.3. Model Method

#### 2.3.1. Design of Optimized Latin Hypercube Experimental Scheme (OLHS)

The main advantage of Latin hypercube sampling lies in its ability to more efficiently cover the sample space, especially in scenarios with non-uniform distributions. Employing a stratified random sampling method ensures that sufficient samples are obtained from different regions of the sample space, thereby avoiding potential bias issues associated with random sampling.

**Figure 3:**
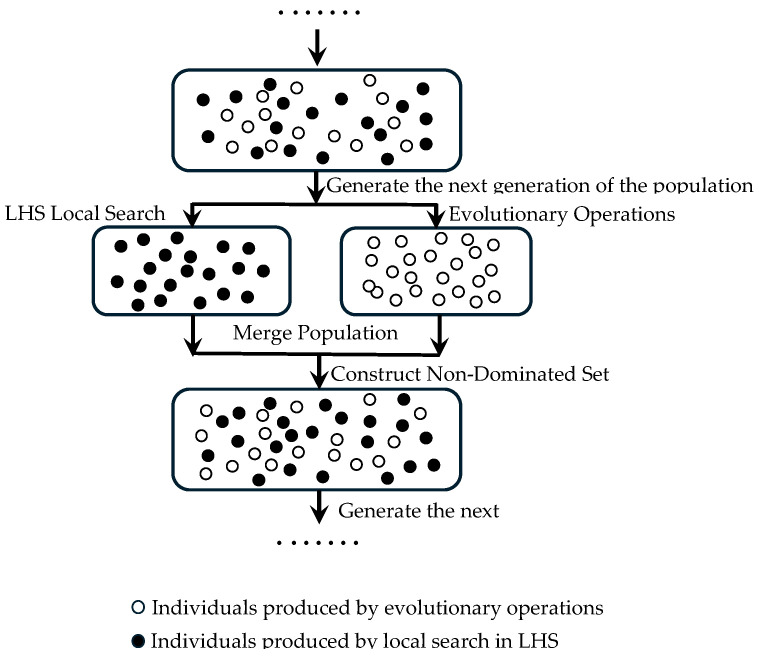
Schematic diagram of the individual-based evolutionary model.

In this manuscript, we propose two evolutionary models, one of which is the individual-based evolutionary model, as illustrated in Figure 3. The characteristic of this model is that each time a parent population is used to produce an offspring population, a certain proportion (p) of individuals from the parent population generate a portion of the offspring population through local search via Latin hypercube sampling (LHS), while the remaining individuals (proportion 1 − p) are generated through evolutionary operations. The evolutionary operations mentioned here include selection, crossover, and mutation operations.

Based on the individual-based evolutionary model, an individual-based LHS-MOEA was designed, and the algorithm is as follows:

Step 1: *t* = 1, parameter initialization. Set the population size as N, the number of evolutionary generations as G, the above proportion parameter as *p*, the neighborhood radius for LHS local search as *δ*, the sampling size as H, the crossover probability as Pc, and the mutation probability as Pm.

Step 2: Initialize the population POP(0).

Step 3: To perform hierarchical ranking on POP(*t*): Rank_Pop(POP(*t*)).

Step 4: *k* = 1.

Step 5: Use the tournament selection mechanism to select an individual *X* from Pop(*t*). If individual *X* is a non-dominated individual (Rank of *X* = 1), then take the neighborhood radius as *δ*; otherwise, take the neighborhood radius as 2*δ*.

Step 6: Generate H individuals using LHS local search within the neighborhood of individual *X* and merge these H individuals into the population: Merge_pop.

Step 7: *k* = *k* + 1.

Step 8: *If k* ≤ *p* × *N*, return to Step 5; otherwise, proceed to Step 9.

Step 9: Use evolutionary operations in Pop(*t*) to generate (1 − *p*) × *N* individuals and merge these individuals into Merge_pop.

Step 10: Implement an elite retention mechanism. Incorporate the parent population Pop(*t*) into the population Merge_pop, i.e., Merge_pop = Merge_Pop ∪ Pop(*t*).

Step 11: To perform hierarchical ranking on the population Merge_pop: Rank_Pop(Merge_pop). Then, use Algorithm 5 to generate a new generation of the population from Merge_pop.

Pop(*t* + 1)New_Pop(Merge_pop).

Step 12: Merge_pop = *Ø*.

Step 13: *If t* ≥ G, then output Pop(*t*) and terminate the algorithm; otherwise, *t* = *t* + 1; return to Step 4 and continue execution.

#### 2.3.2. RBF Neural Network (RBFNN)

**Figure 4:**
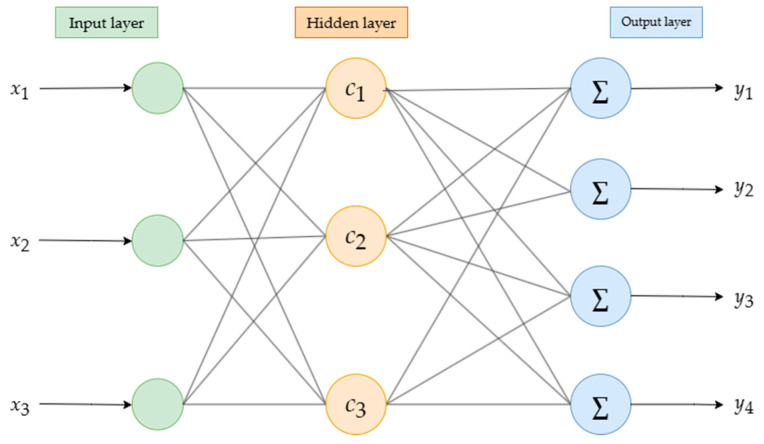
RBFNN model.

RBFNN was proposed by Broomhead and Lowe (1988) based on the principle that biological neurons have local responses. The RBFNN model can utilize a series of discrete data points to fit unknown functions, demonstrating good approximation capabilities for highly complex nonlinear functions, as well as excellent generalization and rapid learning convergence abilities for any nonlinear function, enabling accurate predictions of system behavior. The structure of the RBFNN consists of a three-layer feedforward network. The layer that receives the input signals is the input layer, while the layer that outputs the signals is the output layer, and the layer that is not directly related to the input and output is the hidden layer. A schematic diagram of the RBFNN model is shown in Figure 4.

The RBFNN model consists of two elements. The independent variable is the Euclidean distance between the point to be tested and the sample points, while the basis function is the radial function. In this study, *X* = (*x*_1_, *x*_2_, …, *x*_d_)*^T^* is the d-dimensional input layer vector, *C* = (*c*_1_, *c*_2_, …, *c*_d_)*^T^* is the d-dimensional intermediate hidden layer vector, ‖*X* − *C*‖ is the Euclidean distance between *X* and *C*, *φ* (*X*, *C*, *δ*) is the Gaussian basis function, with input *X* as the variable and center *C* and width *δ* as parameters, and *y* is the output of the neuron. The Gaussian basis function is represented as [21,22].

(5) $$R(x_{p}-c_{i})=exp(-\frac{1}{2\sigma^{2}}{\left\|X-C\right\|}^{2})$$

Here, ${\left\|X-C\right\|}^{2}=\sqrt{\sum_{i=1}^{d}{\left(x_{i}-c_{i}\right)}^{2}}$

Thus, the structure of the radial basis function neural network can obtain the network output as

(6) $$y_{j}=\sum_{i=1}^{h}w_{ij}exp(-\frac{1}{2\sigma^{2}}{\left\|x_{p}-c_{i}\right\|}^{2}),\;j\;=\;1,\;2,\;\ldots ,\;n$$

where *x_p_* is the *p*-th input sample, and *h* is the number of nodes in the hidden layer.

The transformation from the input layer to the hidden layer is a nonlinear transformation that directly maps the input parameters to a new space. The mapping relationship from the hidden layer to the output layer is linear. The environmental parameters for the storage of wolfberries constitute the input layer, establishing a corresponding relationship between the radial basis function neural network and the quality values of the wolfberries. The output layer has four neurons, corresponding to hardness, SSC, TA, and Vc, with different quality values collected from experiments under various storage environments used to train the RBFNN.

#### 2.3.3. Elman Neural Network (ELMAN)

**Figure 5:**
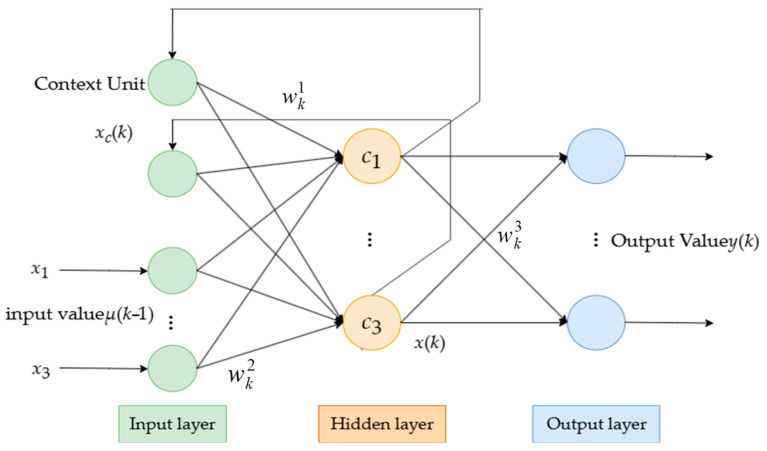
ELMAN.

The Elman recurrent neural network, in addition to the regular input layer, hidden layer, and output layer, includes a special unit known as the context layer or state layer(Figure 5). The input layer units serve the function of signal transmission, while the output layer units perform linear weighting. The hidden layer units typically utilize a nonlinear activation function, and the context layer units receive feedback signals from the hidden layer, specifically to memorize the output values of the hidden layer units from the previous moment, which can be viewed as a one-step delay operator. The input to the network includes not only external input values but also the output values from the previous moment of the hidden layer. At this time, the network can be viewed as a feedforward network and can be trained using the backpropagation algorithm. After the training is completed, the output values of the hidden layer at time k will be fed back to the context layer units through the recurrent connections and retained for the next training instance at time *k* + 1.

Let the external input of the network be *u*(*k* − 1), the output be *y*(*k*), and the output of the hidden layer be *x*(*k*). Then, there is a nonlinear state space expression:

(7) $$x(k)=f[w_{k}^{1}x_{c}(k)+w_{k}^{2}u(k-1)]$$

(8) $$x_{c}(k)=x(k-1)$$

(9) $$y(k)=g[w_{k}^{3}x(k)]$$

In the equation, $w_{k}^{1}$, $w_{k}^{2}$, and $w_{k}^{3}$ represent the connection weight matrices from the context layer to the hidden layer, from the input layer to the hidden layer, and from the hidden layer to the output layer, respectively; *f* and *g* are the transfer functions of the hidden layer and the output layer, respectively.

From Equations (12)–(14), it follows that

(10) $$x_{c}(k)=x(k-1)=f[w_{k-1}^{1}x_{c}(k-1)+w_{k-1}^{2}u(k-2)]$$

Due to the fact that *x*_c_(*k* − 1) = *x*(*k* − 2), the above equation can be further expanded. This indicates that *x*_c_(*k*) depends on the connection weights *w*_1_(*k* − 1), *w*_2_(*k* − 2), …, at different past moments, meaning that *x*_c_(*k*) is a dynamic recursive process. Accordingly, the backpropagation algorithm used for training Elman recurrent neural networks is referred to as the dynamic backpropagation learning algorithm.

#### 2.3.4. Determination of the Optimal Prediction Model

To obtain the optimal prediction model, we evaluate the predictive performance of the constructed model using the root mean square error RMSE and the coefficient of the linear regression line *R*. The calculation method for RMSE is as follows:

(11) $$RMSE=\sqrt{\sum_{j=1}^{n}{\left(P_{j}-M_{j}\right)}^{2}/n}$$

(12) $$R^{2}=1-\sum {\left(M_{j}-P_{j}\right)}^{2}/\sum M_{j}^{2}-\frac{{\left(\sum M_{j}\right)}^{2}}{n}$$

In the equation, *M_j_* and *P_j_* represent the observed value and predicted value of data *j*, respectively, and *n* is the number of observed values.

A good model should be associated with a low RMSE value. Furthermore, if the *R* value is within the range of 0.82 to 0.90, the model demonstrates good performance, while an *R* value above 0.90 indicates that the model is adequate to meet specific prediction objectives.

#### 2.3.5. Particle Swarm Optimization

Particle Swarm Optimization (PSO) is a bionic meta-heuristic algorithm that simulates the social behavior of bird flocks or fish schools. It seeks optimal solutions through cooperation and information sharing among individuals within the swarm. As one of the most classic swarm intelligence algorithms, PSO is widely used to solve single-objective optimization problems due to its simplicity and rapid convergence. Since Moore and Chapman first attempted to extend it to multi-objective optimization [23], the literature has shown that PSO also has significant potential in addressing multi-objective problems [24,25,26].

**Figure 6:**
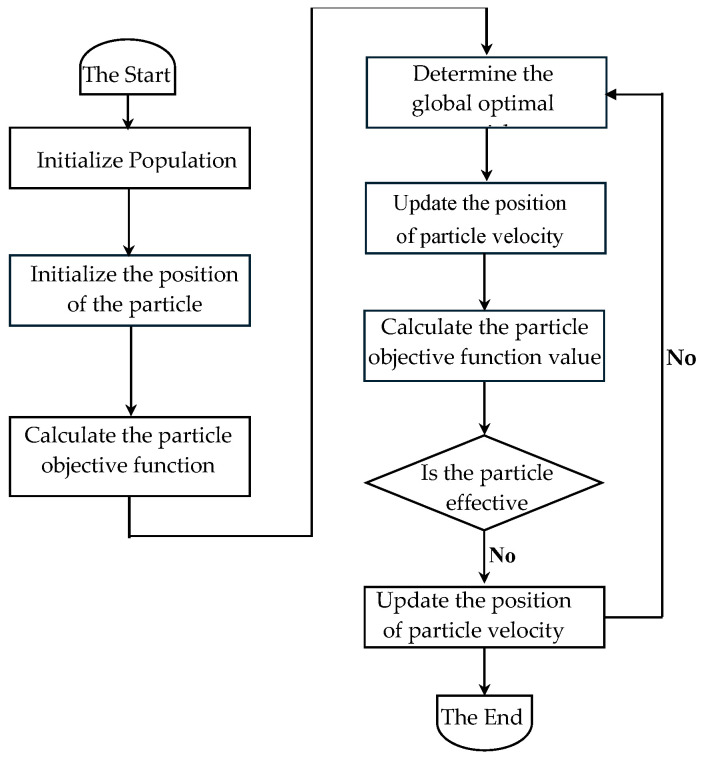
PSO algorithm flowchart.

A.J. Nebro et al. analyzed the “swarm explosion” phenomenon (where the velocity of particles becomes excessively high, resulting in unstable movements of the position limits) faced by mainstream MOPSO algorithms in the literature. They proposed that this issue could be mitigated by implementing a velocity constraint mechanism, leading to the introduction of the velocity-constrained multi-objective particle swarm algorithm SMPSO (Speed-constrained Multi-objective PSO) [27]. Experimental results indicate that, compared to algorithms such as NSGA-II, SPEA2, MOCell, and OMOPSO [28,29,30,31,32,33], SMPSO is capable of handling more complex multi-objective problems (MOPs). The flowchart of the SMPSO algorithm is shown in Figure 6.

## 3. Results

The quality indicators of fresh *Lycium barbarum* in their initial state are (mean ± SD) the following: hardness 18.1 ± 1.20 N, SSC 15.3 ± 1.2%, TA 2.1 ± 0.3%, and Vc 28.5 ± 2.1 mg/100 g.

### 3.1. The Effect of Different Storage Temperatures on the Quality Characteristics of Lycium barbarum *L.*

**Figure 7:**
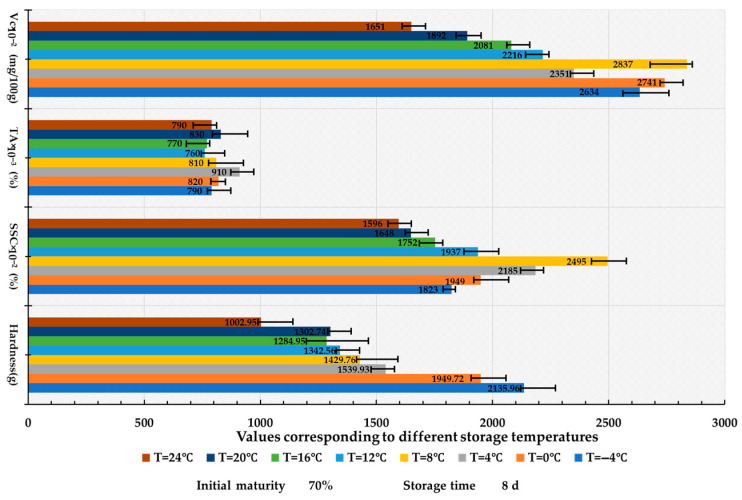
Bar chart of the impact of storage temperature on the quality characteristics of *Lycium barbarum* L.

Fresh *Lycium barbarum* L. with 70% initial maturity and a storage duration of 8 days was utilized for the analysis. The effects of temperature on LBP quality demonstrated significant differences (Figure 7). The soluble solids content (SSC) and vitamin C levels peaked at 8 °C, measuring 24.95% and 28.37 mg/100 g, respectively. Notable parabolic variations were observed, where low temperatures inhibited respiration (Q10 effect) while promoting starch conversion at temperatures between 4 °C and 8 °C (Zolfaghari et al., 2010) [34]. In contrast, increased respiration rates above 8 °C led to diminished nutrient content (Da et al., 2018) [35]. Hardness was found to be maximal at −4 °C due to tissue freezing and exhibited a linear decrease with increasing temperature (R^2^ = 0.93), which was correlated with the degradation of cell wall polysaccharides. The titratable acidity (TA) value remained stable (CV = 4.2%), possibly due to the presence of buffering substances in LBP [2].

### 3.2. The Effect of Different Storage Durations on the Quality Characteristics of Lycium barbarum *L.*

**Figure 8:**
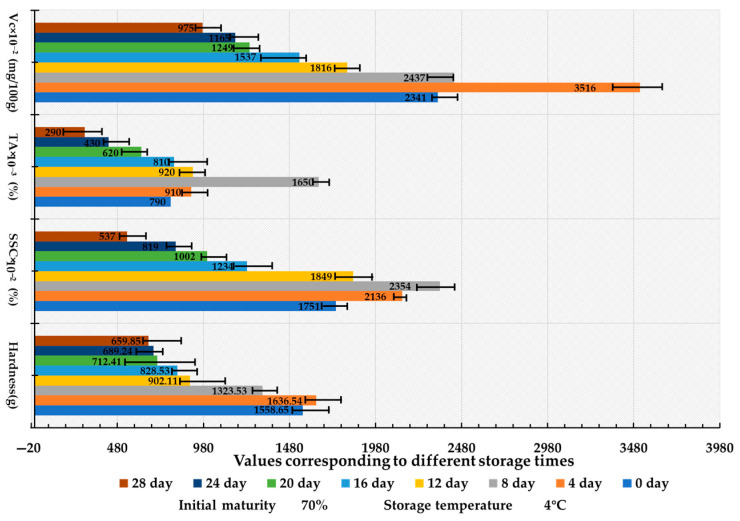
Bar chart of the impact of storage duration on the quality characteristics of *Lycium barbarum* L.

Utilizing an initial maturity of 70% and a storage temperature of 4 °C, the results of the storage test indicated that the influence of storage duration on the quality of fresh *Lycium barbarum* L. exhibited stage-specific characteristics (Figure 8). Notably, hardness and vitamin C (Vc) content peaked on day 4 at 1636.54 g and 35.16 mg/100 g, respectively, which reflects the plant’s response to post-harvest stressors, including cell wall reinforcement and the activation of the L-galactose pathway [36] (Davey et al., 2000). Meanwhile, soluble solids content (SSC) and titratable acidity (TA) reached their maximum values on day 8, measuring 23.54% and 1.65%, respectively, which indicates initial sugar accumulation due to increased amylase activity [34] and subsequent declines later due to respiratory depletion and heightened malate dehydrogenase (MDH) activity. Additionally, microbial action following day 8 resulted in quality deterioration [35].

### 3.3. The Effect of Different Initial Maturity on the Quality Characteristics of Lycium barbarum *L.*

**Figure 9:**
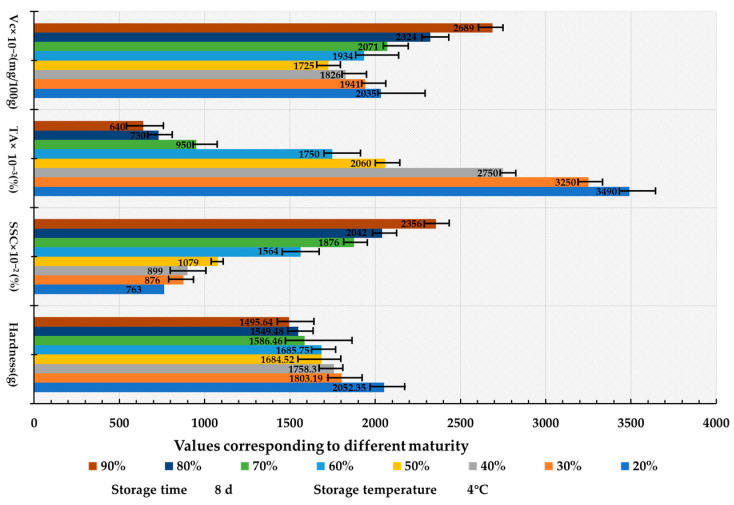
Bar chart of the impact of initial maturity on the quality characteristics of *Lycium barbarum* L.

To investigate the quality evolution mechanism of *Lycium barbarum* L. at various ripeness stages during storage, a storage duration of 8 days at a temperature of 4 °C was established. The change histogram presented in Figure 9 illustrates that both hardness and titratable acidity (TA) decreased synergistically, with hardness decreasing by 27% and TA by 82% in red fruits compared to green fruits. This decline is attributed to reduced malate dehydrogenase (MDH) activity resulting from pectin degradation mediated by polygalacturonase (PGase) and a decrease in the cyclic fluxes of the tricarboxylic acid (TCA) cycle [37]. Furthermore, soluble solids content (SSC) exhibited a significant positive correlation with ripening (R^2^ = 0.91), reaching a peak value of 23.56%, which resulted from a 2.3-fold increase in α-amylase activity in red fruits [38]. Additionally, the osmoregulation of vitamin C (Vc) dynamics displayed a “V-shaped” trend, peaking at 26.89 mg/100 g, indicative of the activation of the L-galactose pathway during the green fruit stage [39], and highlighted the synergistic antioxidant effect of anthocyanins and Vc during the red fruit stage [40]. In conclusion, the ripeness state significantly influenced TA and SSC (*p* < 0.05).

### 3.4. Optimized Latin Hypercube Sampling Experimental Design (OLHS)

The storage process of fresh *Lycium barbarum* L. after harvest is influenced by multiple factors, including the overall storage temperature (T), storage duration (t), and the ripeness of the fruit. Therefore, this study considers three factors as experimental variables: storage temperature (*x*_1_), storage time (*x*_2_), and the ripeness at harvest (*x*_3_), with specific value ranges detailed in Table 1. Based on the optimal Latin hypercube sampling method and the value range of experimental factor levels shown in Table 1, data normalization was performed to standardize the input parameters, as the units of the input parameters are inconsistent, following Equation (13) (as illustrated). When using the LHC matrix for spatial sampling, considering the issue of accuracy, the sample size (n) is calculated using Equation (14), resulting in 40 sets of Latin experimental samples. The target results were obtained through experimentation, as shown in Table 2.

The normalized input parameters are calculated as

(13) $$x=(x_{i}-x_{i\min})/(x_{i\max}-x_{i\min}),i=1,2,3$$

Here, (*i* = 1, 2, 3) represents storage temperature, storage time, and the ripeness of the fruit during storage, respectively.

(14) n=2(N+1)×(N+2)

Here, (*N*) is the number of factors.

#### 3.4.1. Uniformity of Latin Hypercube Sampling

**Figure 10:**
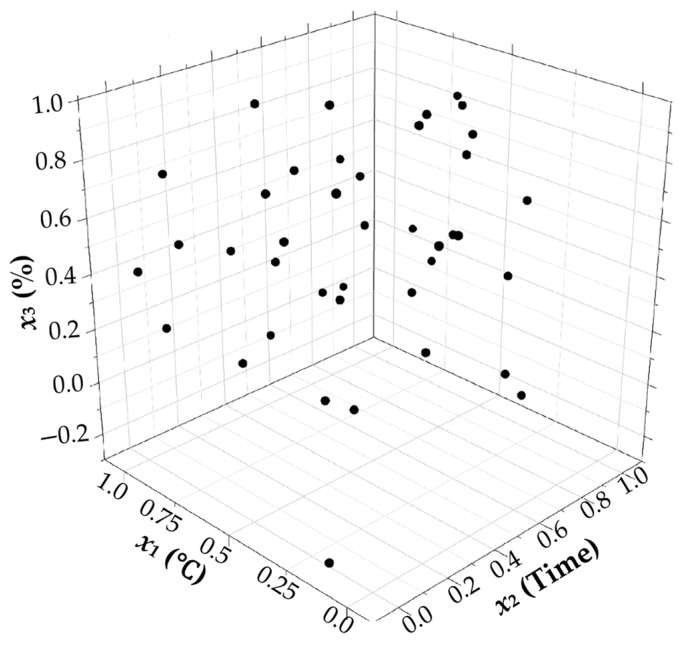
Sampling scatter plot.

**Figure 11:**
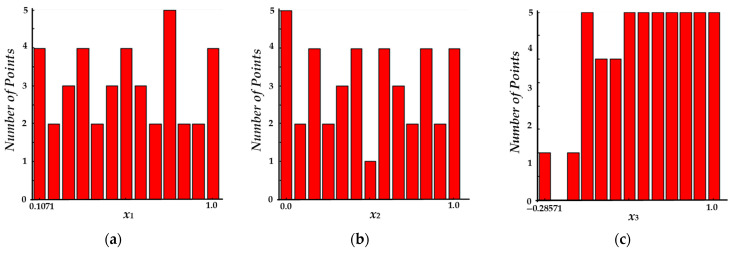
Sampling Frequency Diagram: (**a**) Storage temperature Sampling Frequency Diagram; (**b**) storage time Sampling Frequency Diagram; (**c**) goji berry initial maturity Sampling Frequency Diagram.

Using the optimal Latin hypercube design method, samples of variables *x*_1_, *x*_2_, and *x*_3_ were taken. The sampling diagram is shown in Figure 10, where the sampling points are uniformly distributed in space with no obvious concentration. The uniformity of the values of each factor is also relatively high. As shown in Figure 11, samples are taken in each interval without significant gaps. In summary, the design points generated by the optimal Latin hypercube method are more uniformly distributed in space, leading to improved fitting of design variables and output responses, resulting in more accurate final outcomes.

#### 3.4.2. The Influence of Various Factors on Quality Indicators

**Figure 12:**
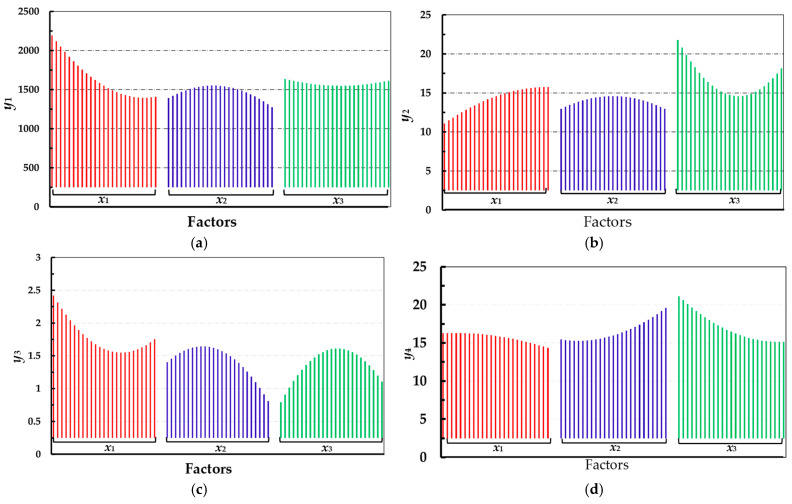
Main Effect Diagrams: (**a**) Main Effect Diagram of *y*_1_; (**b**) Main Effect Diagram of *y*_2_; (**c**) Main Effect Diagram of *y*_3_; (**d**) Main Effect Diagram of *y*_3_.

**Figure 13:**
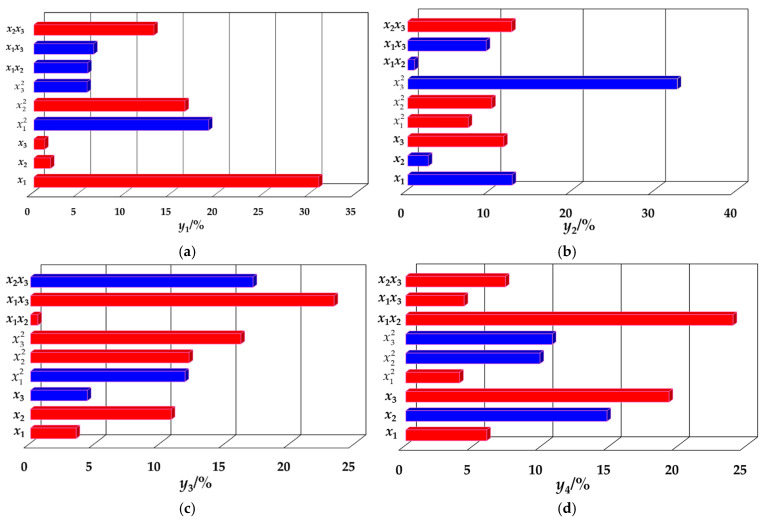
Pareto charts: (**a**) Pareto chart of the effects of various factors on fruit hardness; (**b**) Pareto chart of the effects of various factors on fruit SSC; (**c**) Pareto chart of the effects of various factors on fruit TA; (**d**) Pareto chart of the effects of various factors on fruit Vc.

To analyze the impact of individual factors on quality indicators, this paper conducts a data analysis using a Main Effect Diagram, which shows the average changes in response caused by variations at different levels of each factor. As shown in Figure 12, for the hardness of stored fruit, input variable *x*_2_ and variable *x*_3_ exhibit a trend of extreme values, while variable *x*_1_ shows a monotonically decreasing trend; as shown in Figure 13b, for SSC, variables *x*_2_ and *x*_3_ demonstrate extreme values, and variable *x*_1_ shows a monotonically increasing trend; the three variables have a significant impact on TA, all showing extreme values; for Vc, variables *x*_1_ and *x*_3_ show a monotonically decreasing trend, but it is evident that the decrease in variable *x*_3_ is much greater than that of variable *x*_1_, while variable *x*_2_ exhibits a monotonically increasing trend.

#### 3.4.3. The Correlation Between Storage Environment and the Quality Characteristics of *Lycium barbarum* L.

The Pareto values of each factor are shown in Figure 13a–c. A larger Pareto value indicates a greater proportion of influence of the factor or interaction factor on the evaluation index. The blue bars represent a positive correlation between the factor and the evaluation index, while the red bars indicate a negative correlation.

As shown in Figure 13a, during the storage process, the single factor *x*_1_ has an extremely significant impact on fruit hardness. Among the interaction terms, the interactions of $x_{1}^{2}$, $x_{2}^{2}$, and *x*_2_*x*_3_ have the most significant effect on hardness. Although *x*_1_ contributes significantly to yield, it has a negative contribution value. Additionally, factors such as *x*_2_, *x*_3_, $x_{2}^{2}$, and *x*_2_*x*_3_ are negatively correlated with hardness. Through comprehensive analysis, the influences of *x*_1_, $x_{1}^{2}$, $x_{2}^{2}$, *x*_2_*x*_3_, *x*_1_*x*_3_, *x*_1_*x*_2_, $x_{3}^{2}$, *x*_2_, and *x*_3_ on the hardness of the fruit during storage account for 30.8%, 18.9%, 16.3%, 13.0%, 6.5%, 5.8%, 5.8%, 1.8%, and 1.1%, respectively, indicating that the influence of x3 on hardness is the weakest.

Combining with Figure 13b, it can be observed that the single factors *x*_1_ and *x*_3_ have the most significant impact on SSC, resulting in the largest proportions of 12.668% and 11.64%, respectively. The effect of *x*_2_ on SSC is relatively small, with a proportion of 2.5008%. Among the interaction terms, *x*_2_*x*_3_ has a very significant impact on SSC, with a proportion of 12.618%. The subsequent influences, in descending order, are $x_{2}^{2}$, *x*_1_*x*_3_, $x_{1}^{2}$, and *x*_1_*x*_2_, with corresponding values of 10.194%, 9.522%, 7.3331%, and 0.80928%. Notably, *x*_3_, *x*_2_*x*_3_, $x_{1}^{2}$, and $x_{2}^{2}$ negatively affect SSC.

As shown in Figure 13c, the overall contribution of single factors to TA is relatively small, with the maximum value of *x*_2_ accounting for only 10.823%. The contributions of x1 and *x*_3_ are 3.5044% and 4.3662%, respectively, and both single factors *x*_1_ and *x*_2_ have a negative impact on TA. Among the interaction terms, *x*_1_*x*_3_ has the largest contribution, reaching 23.357%, while the contributions of the other interaction terms *x*_2_*x*_3_, $x_{3}^{2}$, $x_{2}^{2}$, $x_{1}^{2}$, and others are 17.121%, 16.183%, 12.202%, and 11.881%, respectively, with *x*_1_*x*_2_ having the smallest contribution. Notably, *x*_1_*x*_3_, along with other factors, contributes negatively to TA.

The contributions of each single factor and interaction factor to the Vc content during the fruit storage process are shown in Figure 13d. The factors *x*_1_*x*_2_, *x*_3_, *x*_2_, *x*_2_*x*_3_, *x*_1_, *x*_1_*x*_3_, and $x_{1}^{2}$ are negatively correlated with Vc content, with their respective contribution proportions being 23.973%, 19.283%, 14.74%, 7.3001%, 5.9324%, 4.2717%, and 3.9445%.

### 3.5. Goji Berry Quality Indicator Prediction Model

#### 3.5.1. Prediction of Goji Berry Quality Characteristics Based on the RBFNN Regression Prediction Model

**Figure 14:**
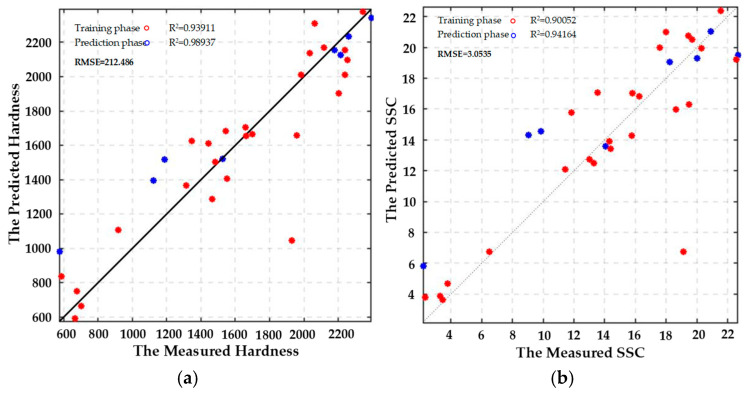

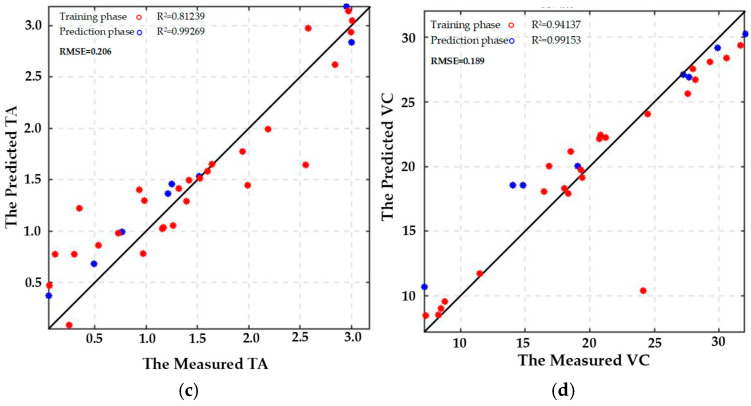
Radial basis function (RBF) neural network regression prediction model: (**a**) Hardness; (**b**) SSC; (**c**) TA; (**d**) Vc.

The RBFNN model was employed for training, with 80% of the data allocated as the training set and 20% as the test set. The number of neurons in the hidden layer was set equal to the number of samples in the training set, totaling 32. Since the network training process is analytical (non-iterative), there is no requirement to define the number of training iterations or termination conditions. The width parameter of the hidden layer is optimized through cross-validation and set to 100 to ensure the balanced performance of the model on both the training and test sets. The analysis of the training results of the RBFNN model indicates that the root mean square errors (RMSEs) for predicting the hardness, SSC, TA, and Vc contents of LBP during storage were 0.24, 0.36, 0.32, and 0.39, respectively, demonstrating the best predictive accuracy with the smallest error. Subsequently, the remaining data were input into the trained model, resulting in four sets of predicted values, as illustrated in Figure 14, with the corresponding R^2^ and RMSE values presented in Table 3. The R^2^ values of the RBFNN model predicting hardness, SSC content, titratable acidity (TA), and vitamin C (Vc) content of *Lycium barbarum* exceeded 0.9, and the RMSE values fell within their respective permissible ranges. Therefore, the developed RBFNN model effectively predicts the relationship between hardness, SSC, TA, and Vc contents under fresh wolfberry storage conditions.

#### 3.5.2. Prediction of Goji Berry Quality Characteristics Based on the ELMAN Regression Prediction Model

**Figure 15:**
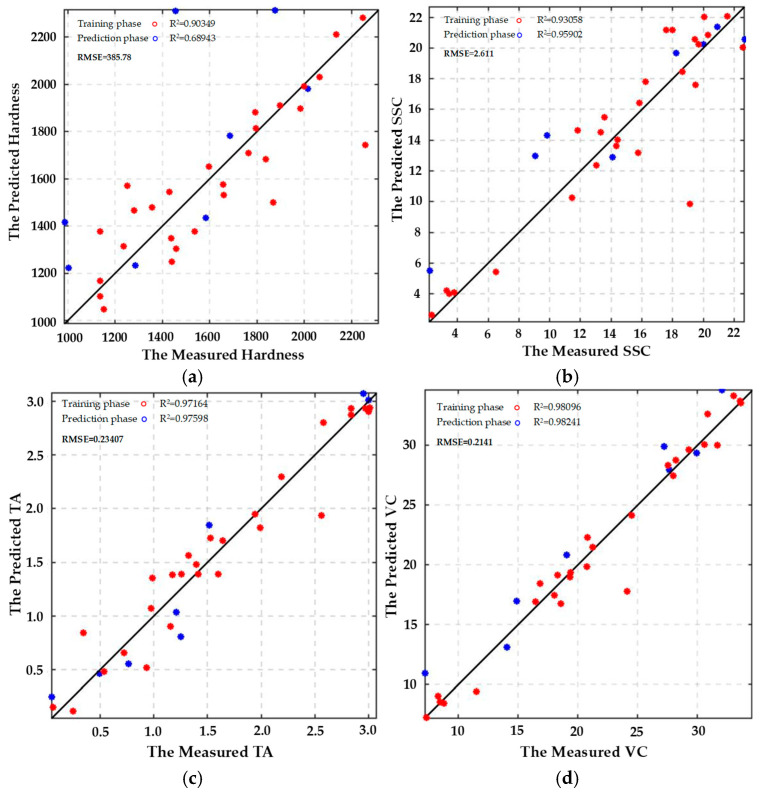
Elman neural network regression prediction model: (**a**) Hardness; (**b**) SSC; (**c**) TA; (**d**) Vc.

The ELMAN model used to predict the quality and texture characteristics of fresh *Lycium barbarum* L. contains a hidden layer with 10 neurons and is trained for 1000 iterations. Training is terminated when the training error falls below 1 × 10^−5^ or the gradient is less than the default threshold of 1 × 10^−6^. The correlation between the measured and predicted values of hardness, SSC, titratable acidity (TA), and vitamin C (Vc) contents of fresh wolfberry during the training and prediction phases of the ELMAN model is presented in Figure 15a–d. The ELMAN model demonstrates higher prediction accuracy for SSC, titratable acidity (TA), and vitamin C (Vc) contents, with corresponding R^2^ values exceeding 0.9. In contrast, the prediction accuracy for hardness during storage is lower, and the specific R^2^ and RMSE values are listed in Table 3. These results indicate that the established ELMAN model effectively predicts the relationships between SSC, TA, and Vc contents and fresh wolfberry storage conditions, but shows poorer performance for predicting changes in hardness during the storage process. Thus, while the model accurately captures the relationship between SSC, TA, and Vc contents and storage conditions, its predictions for hardness changes remain inadequate.

## 4. Multi-Objective Optimization and Experimental Verification

### 4.1. Construction and Solution of the Objective Function

**Figure 16:**
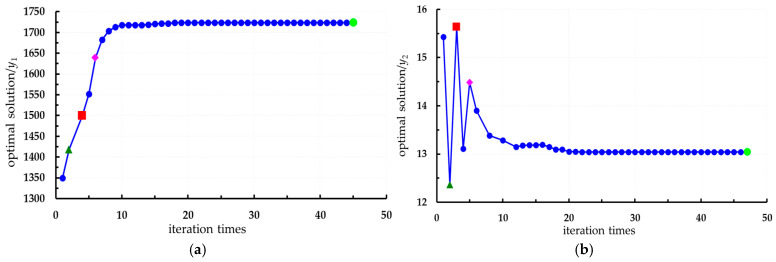

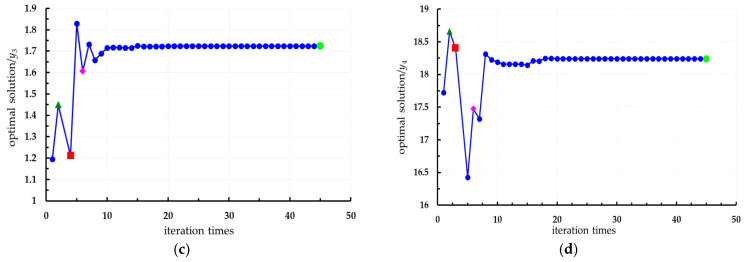
Multi-objective optimization process of quality characteristics: (**a**) Hardness; (**b**) SSC; (**c**) TA; (**d**) Vc.

**Figure 17:**
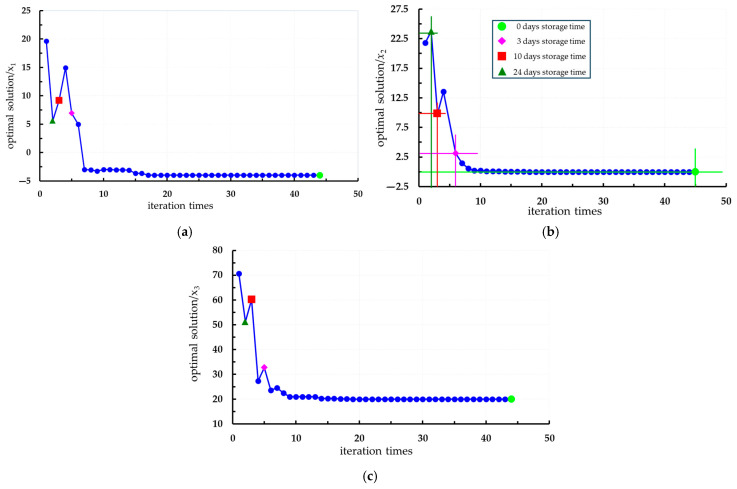
Optimization process of storage conditions: (**a**) Storage temperature; (**b**) storage time; (**c**) initial maturity.

In conjunction with Section 3.5, a regression prediction model for hardness, TA, and Vc was developed using an RBFNN, while a regression prediction model for SSC was constructed using ELMAN with constraints on storage condition parameters, as illustrated in expressions (15) and (16). The indicator weight coefficient serves as a crucial basis for evaluating the quality of fresh *Lycium barbarum* L. during the storage process. In this paper, the weight coefficient is derived from the formula utilized by Zang et al. [39,40,41,42]. The MAPE for each evaluation index oscillates around 5%, indicating high prediction accuracy. Additionally, the NRMSE values for all evaluation indices remain below 5%, suggesting that the prediction model effectively balances various quantitative variables. The SMPSO algorithm was implemented using MATLAB R2021, where the PSO technique was employed to optimize the constructed polynomial equations. An initial population of 12 particles was used over 50 generations of genetic evolution, resulting in a total of 600 selected generations. The optimization aimed for maximum values of hardness, SSC, TA, and Vc, while also deriving quality and storage condition parameters. Following 600 iterations, the optimization process yielded a series of Pareto optimal solution sets, as well as an optimal solution for the four output response variables. The Pareto optimal solution set consists of points that satisfy the optimization criteria, as depicted in Figure 16 and Figure 17. The optimal point was autonomously determined by the computer, represented as the green point in the figure.

(15) $$\left\{\begin{array}{l} y_{Hardness}=2335.8-2578x_{1}+816.4x_{2}-53.13x_{3}+1253.2-864.08+184.21+344.77x_{1}x_{2}+297.58x_{1}x_{3}-533.94x_{2}x_{3}\\ y_{SSC}=11.157+8.859x_{1}+9.233x_{2}-11.724x_{3}-5.8784-6.5147+12.646+0.57922x_{1}x_{2}+5.3007x_{1}x_{3}-6.2718x_{2}x_{3}\\ y_{TA}=1.8548-1.7579x_{1}+0.3851x_{2}+2.1481x_{3}+2.4077-1.9714-1.5817-0.10174x_{1}x_{2}-3.2872x_{1}x_{3}+2.1514x_{2}x_{3}\\ y_{VC}=12.415+10.573x_{1}+9.0241x_{2}-4.5248x_{3}-3.0938+6.1445+4.0587-16.788x_{1}x_{2}-2.3267x_{1}x_{3}-3.5507x_{2}x_{3} \end{array}\right.$$

(16) $$\left\{\begin{array}{c} \max y_{Hardness}(x_{1},x_{2},x_{3})\\ \max y_{SSC}(x_{1},x_{2},x_{3})\\ \max y_{TA}(x_{1},x_{2},x_{3})\\ \begin{array}{l} \max y_{Vc}(x_{1},x_{2},x_{3})\\ s.t.\left\{\begin{array}{l} -4\leq x_{1}\leq 24\\ 0\leq x_{2}\leq 28\\ 20\leq x_{3}\leq 90 \end{array}\right. \end{array} \end{array}\right.$$

From Figure 16 and Figure 17, it can be observed that the optimal storage condition is fresh *Lycium barbarum* L. that have just been harvested (storage time is 0 days), which evidently does not align with actual requirements. Based on the actual storage conditions, different storage durations are selected to determine the storage conditions. Specifically, when stored for 3 days, *Lycium barbarum* L., with an initial maturity of 35%, can be chosen; at a storage temperature of approximately 7.5 °C, the quality characteristics of the *Lycium barbarum* L. are optimal. However, fruits with 35% initial maturity are classified as immature, which does not represent the best harvesting period. At this time, although the values for hardness, SSC, TA, and Vc of the *Lycium barbarum* L. are relatively ideal, they do not meet the harvesting criteria. When stored for 10 days, *Lycium barbarum* L., with an initial maturity of 60%, can be selected, with the storage temperature adjusted to around 10 °C. At this time, the hardness of the *Lycium barbarum* L. is 15 N, the SSC content is 17.5%, the TA content is 1.22%, and the Vc content is 18.5 mg/100 g. When stored for 24 days, *Lycium barbarum* L., with an initial maturity of 50%, are selected, and the temperature is adjusted to around 5 °C. At this time, the hardness of the fruit is 1425, the SSC content is 12.45%, the TA content is 1.45%, and the Vc content is 18.75 mg/100 g. In summary, it is advisable to choose *Lycium barbarum* L. with an initial maturity of over 60%, with a storage duration of around 20 days, and a storage temperature adjusted to 10 °C, as the optimal storage conditions.

### 4.2. Experimental Verification

Fresh *Lycium barbarum* L. harvested were stored in a temperature-controlled chamber at 10 °C, with testing conducted in three batches over a period of two months. The quality characteristics of the wolfberries were assessed at 24 days of storage. By transforming and analyzing the measured data, we conclude that the hardness of goji berry fruit is 13.85 N, the SSC is 11.06%, the TA is 1.38%, and the Vc content is 18.23 mg/100 g. These values fall within the error range when compared to the estimates derived from the prediction model constructed in Section 4.1, thereby demonstrating that the prediction model can be considered effective.

In conclusion, the RBFNN model developed for predicting the quality characteristics of fresh *Lycium barbarum* L. can provide theoretical guidance for establishing optimal storage conditions for various fresh fruits, including cherries, peaches, and other perishable varieties. This model is based on an optimized Latin hypercube sampling methodology (Table 4).

## 5. Conclusions

(1) This study systematically elucidates the differential effects of temperature, duration, and initial maturity on the storage quality of fresh *Lycium barbarum* L. The findings indicated that storage temperature significantly influenced fruit hardness (contributing up to 30.8%), (SSC), and Vc content, while exerting a comparatively minor effect on TA. Additionally, storage duration exhibited a characteristic trend of initially increasing and then decreasing SSC, TA, and Vc contents, whereas hardness consistently decreased with extended storage time. Notably, harvest initial maturity exerted the most significant regulatory effect on TA and SSC contents, contributing 19.28%, while its effects on hardness and Vc contents were relatively limited.
(2) This study established a comprehensive analytical framework. An optimal Latin hypercube experimental design was employed to ensure uniform distribution of sampling points within the multidimensional parameter space. Utilizing the Pareto analysis method, we quantified, for the first time, the contribution of each storage condition to the quality indices, revealing that *x*_1_ had the most significant impact on hardness, while *x*_3_ contributed the most to Vc. Additionally, we innovatively developed a radial basis RBFNN prediction model, which demonstrated a marked improvement in prediction accuracy compared to ELMAN, achieving a 35% reduction in error, particularly in hardness prediction.
(3) Through the application of the PSO algorithm for multi-objective optimization, the optimal combination of storage parameters was determined: a storage temperature of 10 °C, a storage time of 20 days, and a harvest initial maturity of ≥60%. Under these optimized conditions, the quality indices of fresh *Lycium barbarum* L. reached an optimal balance: hardness was measured at 15.01 N, SSC was 17.5%, TA was 1.22%, and Vc content was 18.5 mg/100 g.
(4) The whole-chain analysis framework of “experimental design-machine learning-intelligent optimization” established in this study not only offers a scientific decision-making method for the preservation of fresh *Lycium barbarum* L., but also provides a technical pathway for quality control research of other distinctive agricultural products. Nonetheless, the analytical process is limited by challenges such as product homogenization, extreme storage conditions (e.g., temperatures exceeding 12 °C or initial maturity levels surpassing 80%), and insufficient integration of biological mechanisms. To enhance the model’s accuracy, we will incorporate mechanistic models (e.g., the Michaelis–Menten equation) to develop a hybrid model, broaden the variety of samples, and increase the volume of real-time monitoring data from near-infrared spectroscopy in future research.

In summary, while a value of R^2^ >0.94 on the test sets indicates robustness, future work will validate against external datasets to ensure generalization beyond Gansu-sourced berries, and future studies will validate the model across multiple harvest seasons and expand applications to commercial supply chains.

## Figures and Tables

**Table 1:** Experimental factors and their value ranges.

Input Factors	Storage Temperature (°C)	Storage Time (Days)	Initial Maturity (%)
Value range	−4 to 24	0 to 28	20 to 90

**Table 2:** Optimized Latin hypercube experimental design.

Serial Number	Influencing Factors			Quality Indicators			
	*x*_1_	*x*_2_	*x*_3_	*y*_1_	*y*_2_	*y*_3_	*y*_4_
				Hardness (N)	SSC (%)	TA (%)	Vc (mg/100 g)
1	0.66	0.51	0.18	14.08	6.25	1.76	22.23
2	0.68	0.62	0.49	12.56	10.36	2.34	15.34
3	0.27	0.13	0.15	19.76	9.12	4.12	17.36
4	0.91	0.85	0.36	13.30	8.45	2.19	16.28
5	0.77	0.03	0.23	22.15	16.59	0.82	18.25
6	0.31	0.28	0.97	11.13	20.16	0.98	22.16
7	0.63	0.69	0.82	9.67	19.26	0.71	17.52
8	0.50	1.00	0.85	9.84	6.15	0.75	12.35
9	0.20	0.92	0.77	14.28	14.61	0.49	26.42
10	0.79	0.77	0.05	18.33	12.34	0.95	14.65
11	0.29	0.97	0.31	20.94	5.36	1.03	21.35
12	0.70	0.95	0.59	15.06	10.57	1.26	18.46
13	0.95	0.64	0.64	12.12	14.46	1.09	14.52
14	0.22	0.08	0.74	18.59	15.26	0.42	16.79
15	0.11	0.44	0.26	24.21	5.43	3.43	9.36
16	0.75	0.36	0.69	14.31	11.52	1.12	12.52
17	0.13	0.41	0.72	22.03	16.85	0.36	19.56
18	0.47	0.77	0.00	16.26	14.36	2.42	11.54
19	0.84	0.33	0.38	15.53	13.42	1.34	17.56
20	0.24	0.23	0.46	18.40	12.46	0.97	17.42
21	0.79	0.10	0.90	14.02	21.35	1.52	21.52
22	0.98	0.56	0.21	11.30	17.42	1.03	13.26
23	0.43	0.82	0.56	17.60	19.54	1.52	16.52
24	0.38	0.59	0.33	18.00	11.26	1.95	14.26
25	0.15	0.72	0.51	19.46	15.62	2.75	21.36
26	0.40	0.49	0.67	17.31	18.43	0.46	18.42
27	0.57	0.21	0.10	15.65	17.26	0.95	8.79
28	0.61	0.38	1.00	12.60	21.03	0.25	28.42
29	0.59	0.87	0.28	9.63	19.42	2.15	7.69
30	0.52	0.15	0.77	11.14	17.03	1.26	18.79
31	0.86	0.90	0.87	8.01	18.06	0.85	21.52
32	0.73	0.05	0.54	12.27	20.71	3.41	12.89
33	0.54	0.31	0.44	16.25	12.36	0.95	19.52
34	1.00	0.18	0.62	11.14	21.59	1.09	18.42
35	0.45	0.00	0.41	16.53	15.62	0.56	15.49
36	0.34	0.67	0.92	17.57	13.52	0.26	11.03
37	0.93	0.26	0.08	14.11	11.34	1.96	16.85
38	0.36	0.46	0.03	20.25	21.52	2.63	18.42
39	0.89	0.54	0.95	8.97	19.53	1.65	12.85
40	0.18	0.74	0.13	19.61	15.85	2.09	18.04

Note: The values in the center of the table are the theoretical design values for Latin hypercube sampling that were fine-tuned during the actual experiments based on the accuracy of the equipment.

**Table 3:** The coefficients of determination (R^2^) and root mean square error (RMSE) of various models for the evaluation indicators during the prediction and training phase are presented.

Model	Phase	Indicators of Predictive Accuracy	Hardness	SSC	TA	Vc
RBFNN	Training	R^2^	0.99	0.97	0.99	0.99
		RMSE	185.21	2.10	0.18	0.16
	Prediction	R^2^	0.98	0.94	0.99	0.99
		RMSE	212.49	3.05	0.21	0.19
ELMAN	Training	R^2^	0.85	0.98	0.99	0.99
		RMSE	310.75	1.85	0.20	0.18
	Prediction	R^2^	0.69	0.96	0.98	0.98
		RMSE	385.78	2.611	0.23407	0.2141

**Table 4:** Standardized error metrics are essential for the evaluation of prediction models.

Indicators of Predictive Accuracy	Hardness (%)	SSC (%)	TA (%)	Vc (mg/100 g)
MAPE	6.2	4.8	7.5	5.9
NRMSE	0.18	0.15	0.21	0.16

## Data Availability

The original contributions presented in this study are included in the article. Further inquiries can be directed to the corresponding authors.

## References

[1] Chinese Pharmacopocia Commissiom (2015). Chinese Pharmacopocia, Beijing, China.

[2] Sun W., Shahrajabian M.H., Cheng Q. (2021). Health benefits of wolfberry (Gou Qi Zi, *Fructus barbarum* L.) on the basis of ancient Chineseherbalism and Western modern medicine. Avicenna J. Phytomed.

[3] Emamgholizadeh S., Parsaeian M., Baradaran M. (2015). Seed yield prediction of sesame using artificial neural network. Eur. J. Agron..

[4] Torkashvand A.M., Ahmadi A., Nikravesh N.L. (2017). Prediction of kiwifruit firmness using fruit mineral nutrient concentration by artificial neural network (ANN) and multiple linear regressions (MLR). J. Integr. Agric..

[5] Zhang J., Chen C., Wu C., Kou X., Xue Z. (2024). Storage quality prediction of winter jujube based on particle swarm optimization-backpropagation-artificial neural network (PSO-BP-ANN). Sci. Hortic..

[6] Xing W., Liu W., Li H., Zeng X., Fan X., Xing S., Gong H. (2025). Development of predictive models for shelf-life of sweet cherry under different storage temperatures. LWT.

[7] Zhang Y., Zhu D., Ren X., Shen Y., Cao X., Liu H., Li J. (2022). Quality changes and shelf-life prediction model of postharvest apples using partial least squares and artificial neural network analysis. Food Chem..

[8] Niazian M., Sadat-Noori S.A., Abdipour M. (2018). Modeling the seed yield of Ajowan (*Trachyspermum ammi* L.) using artificial neural network and multiple linear regression models. Ind. Crop. Prod..

[9] Mohammed M., Munir M., Aljabr A. (2022). Prediction of Date Fruit Quality Attributes during Cold Storage Based on Their Electrical Properties Using Artificial Neural Networks Models. Foods.

[10] Huang X., Chen T., Zhou P., Huang X., Liu D., Jin W., Zhang H., Zhou J., Wang Z., Gao Z. (2022). Prediction and optimization of fruit quality of peach based on artificial neural network. J. Food Compos. Anal..

[11] Tracey J., Zhu J., Crooks K.R. (2011). Modeling and inference of animal movement using artificial neural networks. Environ. Ecol. Stat..

[12] Lan H., Wang Z., Niu H., Zhang H., Zhang Y., Tang Y., Liu Y. (2020). A nondestructive testing method for soluble solid content in Korla fragrant pears based on electrical properties and artificial neural network. Food Sci. Nutr..

[13] Sanad Alsbu R.A., Yarlagadda P., Karim A. (2023). An Empirical Model for Predicting the Fresh Food Quality Changes during Storage. Foods.

[14] Bajd F., Škrlep M., Čandek-Potokar M., Serša I. (2017). MRI-aided texture analyses of compressed meat products. J. Food Eng..

[15] Owoyemi A., Balaklav M., Kochanek B., Porat R., Koenigstein N., Salzer Y., Lichter A. (2024). Deviations from optimal storage temperature and its impact on postharvest quality of table grape cv. Scarlotta Seedless. Postharvest Biol. Technol..

[16] Lin F., Chen D., Liu C., He J. (2024). Non-Destructive Detection of Golden Passion Fruit Quality Based on Dielectric Characteristics. J. Appl. Sci..

[17] Jakhar V., Baswal A.K., Gupta A., Gill K.S., Ozturk B. (2023). Impact of Post-harvest Application of Methyl Salicylate and Salicylic Acid on Storage Life and Quality of ‘Kinnow’ Mandarin (*Citrus nobilis* Lour × *C*. *deliciosa* Tenora) Fruit Under Ambient Storage Conditions. Erwerbs-Obstbau.

[18] Andrea C.G.S., Angel G., Mari I.G. (2003). Comparative study of six pear cultivars in terms of their phenolic and vitamin C contents and antioxidant capacity. J. Sci. Food Agric..

[19] Wang Z., Wang W., Jiang Y., Bao A., Tong W., Wang B. (2020). Effects of different harvesting periods on the storage quality and senescence of apple at room temperature. Trans. CSAE.

[20] (2016). National Food Safety Standard—Determination of ascorbic acid in foods.

[21] Zeng X., Zhen Z., He J., Han L. (2018). A feature selection approach based on sensitivity of RBFNNs. Neurocomputing.

[22] Hu Y., You J., Liu J., He T. (2018). An eigenvector based center selection for fast training scheme of RBFNN. Inf. Sci..

[23] Moore J., Chapman R. (1999). Application of Particle Swarm to Multiobjective Optimization.

[24] Coello Coello C.A., Lecsuga M.S. MOPSO: A proposal for multiple objective particle swarm optimization. Proceedings of the IEEE Congress on Evolutionary Computation.

[25] Dai C., Wang Y., Ye M. (2015). A new multi-objective particle swarm optimization algorithm based on decomposition. Inf. Sci..

[26] Zhang X., Zheng X., Cheng R., Qiu J., Jin Y. (2018). A competitive mechanism based multi-objective particle swarm optimizer with fast convergence. Inf. Sci..

[27] He C., Li M., Zhang C., Chen H., Li X., Li J. (2022). A competitive swarm optimizer with probabilistic criteria for many-objective optimization problems. Complex Intell. Syst..

[28] Durillo J.J., García-Nieto J., Nebro A.J., Coello C.A.C., Luna F., Alba E. (2009). Multi-objective particle swarm optimizers: An experimental comparison. Proceedings of the 5th International Conference on Evolutionary MultiCriterion Optimization.

[29] Nebro A.J., Durillo J.J., Garcia Nieto J., Coello Coello C.A., Luna F., Alba E. SMPSO: A new PSO-based metaheuristic for multiobjective optimization. Proceedings of the IEEE Symposium on Computational Intelligence in Miulti-Criteria Decision-Making.

[30] Deb K., Pratap A., Agarwal S., Meyarivan T. (2002). A fast and elitist multiobjective genetic algorithm: NSGA-II. IEEE Trans. Evol. Comput..

[31] Zitzler E., Laumanns M., Thiele L. (2001). SPEA2: Improving the Strength Pareto Evolutionary Algorithm.

[32] Nebro A.J., Durillo J.J., Luna F., Dorronsoro B., Alba E., Obayashi S., Deb K., Poloni C., Hiroyasu T., Murata T. (2007). Design issues in a multiobjective cellular genetic algorithm. Proceedings of the Evolutionary Multi-Criterion Optimization, 4th International Conference, EMO 2007.

[33] Reyes Sierra M., Coello Coello C.A., Coello Coello C.A., Hernández Aguirre A., Zitzler E. (2005). Improving PSO-Based Multi-Objective Optimization Using Crowding, Mutation and ε-Dominance. Proceedings of the Evolutionary Multi-Criterion Optimization: Third International Conference, EMO 2005.

[34] Zolfaghari M., Sahari M.A., Barzegar M., Samadloiy H. (2008). Physicochemical and enzymatic properties of five kiwifruit cultivars during cold storage. Food Bioprocess Technol..

[35] Da Y., Li D., Xu W., Fu W., Liao R., Shi J., Wang J. (2018). Effects of packaging design with dual function films on quality of wax apples stored at ambient temperatures. Food Bioprocess Technol..

[36] Davey M.W., Van Montagu M., Inze D., Sanmartin M., Kanellis A.K., Smirnoff N., Benzie J., Strain J., Favell D., Fletcher J.M. (2020). Plant L-ascorbic acid: Chemistry, function, metabolism, bioavailability and effects of processing. J. Sci. Food Agric..

[37] Li X., Yang Y., Li M., Chen Y., Li W., Zhou L., Sun X., Wang C., Chen X. (2022). Dynamics of Organic Acids in Goji Berry (*Lycium barbarum* L.) During Postharvest Storage. Food Chem..

[38] Zhang R., Liu Y., Zhao X., Zhang Y., Zhou X., Sun X., Wang C., Chen X. (2023). Starch degradation enzymes in relation to soluble sugars accumulation during goji berry ripening. Postharvest Biol. Technol..

[39] Wang Y., Zhao X., Liu Y., Zhang W., Zhang R., Hu P., Wang W., Li Y., Zhang R. (2018). Anthocyanin accumulation and molecular analysis of correlated genes in *Lycium ruthenicum* Murray. Acta Physiol. Plant..

[40] Zang Z., Huang X., He C., Zhang Q., Jiang C., Wan F. (2023). Improving Drying Characteristics and Physicochemical Quality of Angelica sinensis by Novel Tray Rotation Microwave Vacuum Drying. Foods.

[41] Zang Z., Huang X., Ma G., Zhang Q., Jiang C. (2022). Evaluation of drying characteristics and physicochemical properties of *Angelicae sinensis* Radix under different drying methods based on combination entropy weight and variable coefficient method. Chin. Tradit. Herb. Drugs.

[42] Zang Z., Wan F., Ma G., Xu Y., Wu B., Huang X. (2024). Effect of ultrasound combined with chemical pretreatment as an innovative non-thermal technology on the drying process, quality properties and texture of cherry subjected to radio frequency vacuum drying. Ultrason. Sonochemistry.

